# Supplementary material for: Gibberellic Acid Modifies the Transcript Abundance of ABA Pathway Orthologs and Modulates Sweet Cherry (Prunus avium) Fruit Ripening in Early- and Mid-Season Varieties
Source: Plants (Basel). 2020 Dec 18;9(12):1796. doi: 10.3390/plants9121796 (PMC7767171; doi:10.3390/plants9121796)
Supplement: Supplementary file 1 [file plants-09-01796-s001.zip › Supplementary Material.docx]

**Gibberellic acid modifies the transcript abundance of ABA pathway orthologs and modulates sweet cherry (*Prunus avium*) fruit ripening in early- and mid-season varieties.**

**Supplementary** **Material**

**Supplementary Figures**


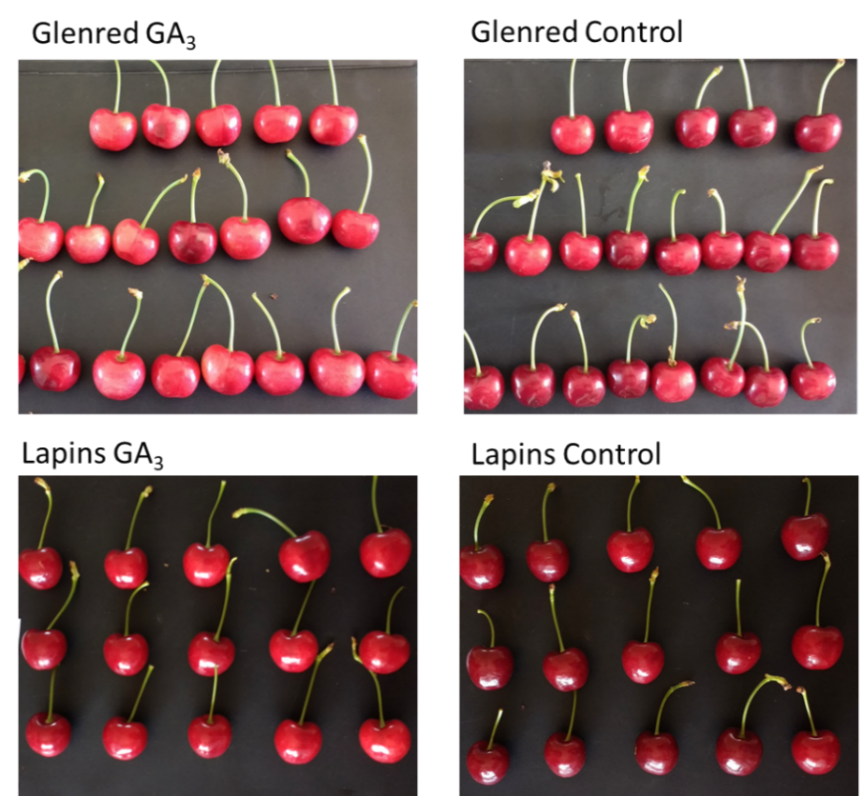


Figure S1: Effect of GA_3_ on fruit color the 2017-2018 season. Representative picture of control and GA_3_-treated fruits at harvest (59 DAFB, 10^th^ November, 2017, in Glenred; 69 DAFB, 29^th^ November 2017, in Lapins).





Figure S2: Effect of GA_3_ on IAD of early-season variety, Glenred (A) and mid-season variety, Lapins (B) in the 2018-2019 season 20 fruits from control and GA3 trees of Lapins and Glenred, were randomly selected for nondestructive IAD measurements in the field m Data as ± SEM ANOVA with Tukey’s *posthoc* test at p < 0 05 was conducted; ‘*’ denotes statistical differences between GA_3_-treated and control fruits at the different dates DAFB days after full bloom


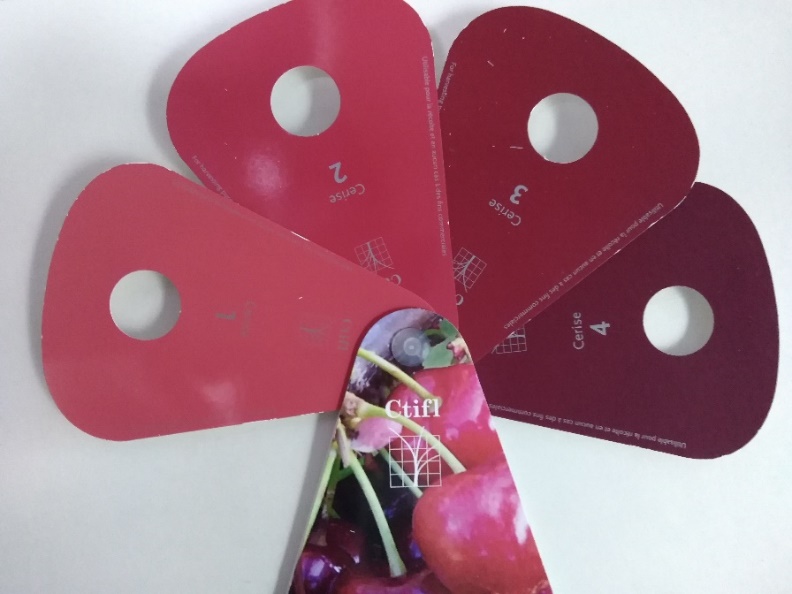


Figure S3: CTIFL color chart. In this study 1 to 4 color categories were used.

**Supplementary Tables**

Table S1: Fruit color phenology of early-variety Glenred obtained during 2017-2018 season.

| Days after full bloom (DAFB) | Date | Phenology |
| --- | --- | --- |
| 14 | September 26^th^, 2017 | 90% green and 10% light green |
| 20 | October 2^nd^, 2017 | 10% green and 90% light green |
| 27 | October 9^th^, 2017 | 5% yellow and 95% light green |
| 31 | October 13^th^, 2017 | 10% yellow and 90% light green |
| 36 | October 18^th^, 2017 | 40% yellow and 60% light green |
| 41 | October 23^rd^, 2017 | 10% yellow with pink blushes and 90% yellow |
| 45 | October 27^th^, 2017 | 25% yellow with pink blushes and 75% yellow |
| 51 | November 2^nd^, 2017 | 10% yellow with pink blushes 60% pink and 30% red |
| 56 | November 7^th^, 2017 | 20% pink, 40% pink with red blush and 40% red |
| 59 | November 10^th^, 2017 | 20% pink with red blush and 80% red |
| 64 | November 24^th^, 2017 | 70% red and 30% mahogany |

Table S2: Fruit color phenology of mid-variety Lapins during 2017-2018 season.

| Days after full bloom (DAFB) | Date | Phenology |
| --- | --- | --- |
| 16 | October 7^th^, 2017 | 90% green and 10% light green |
| 20 | October 11^th^, 2017 | 70% green and 30% light green |
| 24 | October 15^th^, 2017 | 50% green and 50% light green |
| 29 | October 20^th^, 2017 | 1% yellow and 99% light green |
| 33 | October 7^th^, 2017 | 10% yellow and 90% light green |
| 37 | October 26^th^, 2017 | 20% yellow and 80% light green |
| 43 | November 3^rd^, 2017 | 30% yellow and 70% light green |
| 49 | November 9^th^, 2017 | 50% yellow and 50% light green |
| 56 | November 17^th^, 2017 | 10% yellow with pink blushes and 90% yellow |
| 61 | November 21^th^, 2017 | 25% yellow with pink blushes and 75% yellow |
| 65 | November 25^th^, 2017 | 10% yellow with pink blushes 70% pink and 20% red |
| 68 | November 28^th^, 2017 | 50% pink and 50% red |
| 69 | December 1^st^, 2017 | 20% pink, 20% pink with red blushed and 60% red |
| 74 | December 4^th^, 2017 | 80% red and 20% mahogany |

Table S3: Primers used for RT-qPCR gene expression analyses in sweet cherry fruits. F: Forward primer; R: reverse primer; Tm: melting temperature.

| Gene symbol | Primer Name | Primer Nucleotidic Sequence | Tm ºC | Reference |
| --- | --- | --- | --- | --- |
| *PavNCED1* | PacNCED1-F | 5’ - CTCCAGAGTTCCGTATGGTTTTC - 3’ | 59,07 | Li et al. 2015a |
|  | PacNCED1-R | 5’ - TAGCTTCCCACAGGTAATTGTCC - 3’ | 60,05 |  |
| *CYP707A2* | PacCYP707A2-F | 5’ - GAACAATCACCACCACAAAGAACTG - 3’ | 60,96 | Li et al. 2015a |
|  | PacCYP707A2-R | 5’ - CTTGCCGAGACCGATTTATTGTATG - 3’ | 60,33 |  |
| *PP2C3* | PacPP2C3-F | 5’ - TGGAGTTTTCCGGCTCTTCC - 3’ | 59,96 | This study* |
|  | PacPP2C3-R | 5’ - TCGGCAAAACGATGGGTGTA - 3’ | 59,96 |  |
| *PP2C4* | PacPP2C4-F | 5’ - GGAGTCTAAATCTGTGCGTGTGGAC - 3’ | 63,55 | Wang et al. 2015 |
|  | PacPP2C4-R | 5’ - AAACTCCATAGAAGTGGGCTCCATT - 3’ | 62,06 |  |
| *SnRK2.1* | PacSnRK2.1-F | 5’ - ATTGTAGGCATCTGCTCTCTCGCAT - 3’ | 64,21 | Wang et al. 2015 |
|  | PacSnRK2.1-R | 5’ - TGAGCTGCTTCCGTAAGCTCTCTAG - 3’ | 63,88 |  |
| *SnRK2.2* | PacSnRK2.2-F | 5’ - AAACTATGCGGCAGTGGAGCG - 3’ | 63,60 | Wang et al. 2015 |
|  | PacSnRK2.2-R | 5’ - ACATCGACTTCGGAGTCCAAATCAG - 3’ | 62,85 |  |
| *SnRK2.3* | PacSnRK2.3-F | 5’ - GTTGCTACAGCAATGGAGCG - 3’ | 59,60 | This study* |
|  | PacSnRK2.3-R | 5’ - TGGGATGCTTCAAGGACCTG - 3’ | 59,67 |  |
| *CAC* | PavCAC-F | 5’ - GCGTGCCAGACTGACCTT - 3’ | 59,97 | Alkio et al. 2012 |
|  | PavCAC-R | 5’ - GGCGAGCGTGACATATCTAACC - 3’ | 61,17 |  |
| *TEF2* | PavTEF2-F | 5’ - TGAAGGAGAGGGAAGGTGAAAG - 3’ | 55,50 | Tong et al. 2009 |
|  | PavTEF2-R | 5’ - GGTGTGACGATGAAGAGTGATG - 3’ | 56,30 |  |
| *ACT1* | PavACT1-F | 5’ - CTCCTCTCAACCCTAAGGCTAACAG - 3’ | 67,40 | Wang et al 2015 |
|  | PavACT1-R | 5’ - CAGTTGTACGACCACTGGCATACAG - 3’ | 67,40 |  |

* Primers redesigned for the same template sequence reported in Wang et al. (2015).
