# Supplementary figures and images for: Gibberellic Acid Modifies the Transcript Abundance of ABA Pathway Orthologs and Modulates Sweet Cherry (Prunus avium) Fruit Ripening in Early- and Mid-Season Varieties
Source: Plants (Basel). 2020 Dec 18;9(12):1796. doi: 10.3390/plants9121796 (PMC7767171; doi:10.3390/plants9121796)

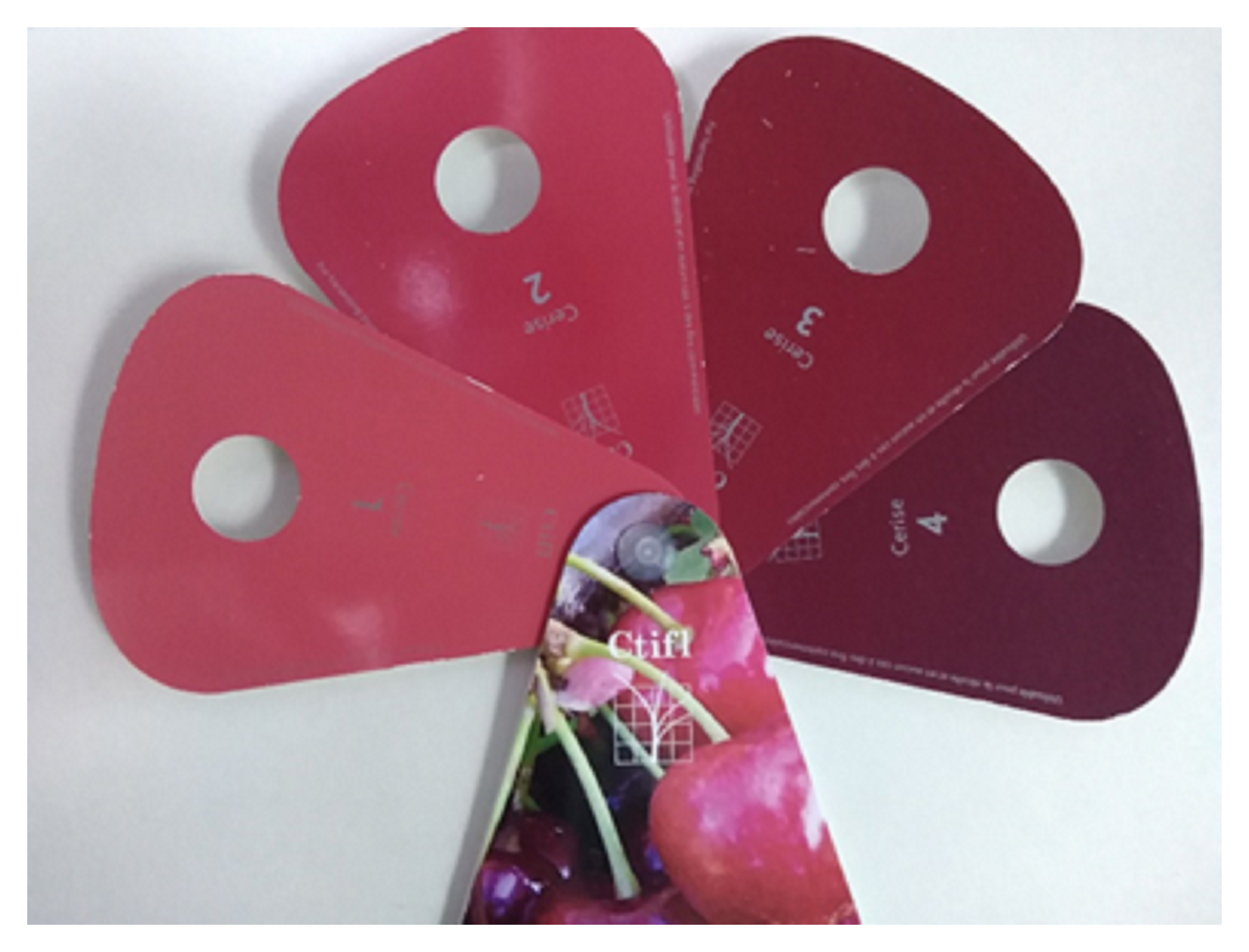

Supplement: Supplementary file 1 [file plants-09-01796-s001.zip › FIGURE SUPP 3.tif]

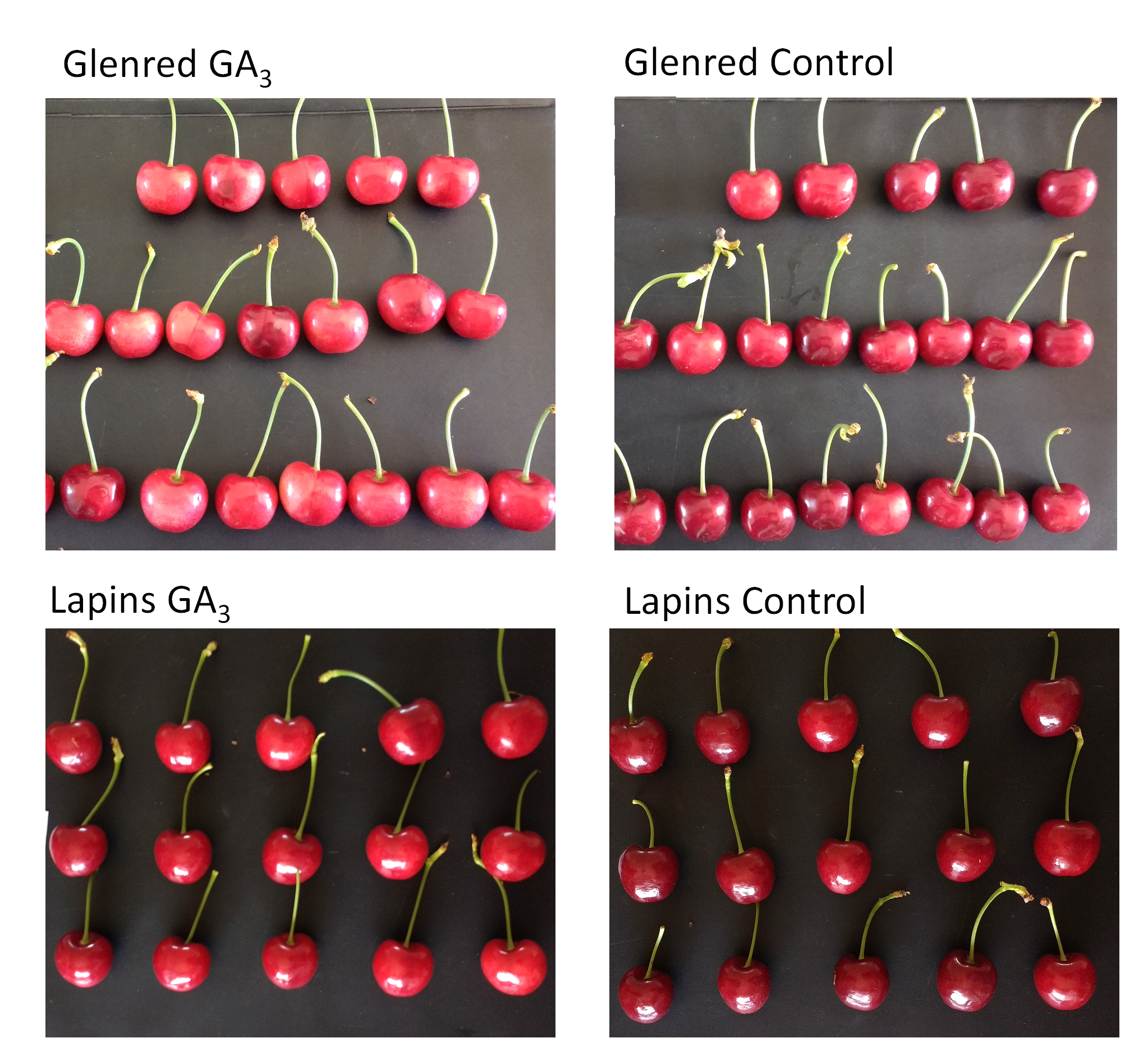

Supplement: Supplementary file 1 [file plants-09-01796-s001.zip › FIGURE SUPP 1.tif]
